# Quantitative assessment of the stent/scaffold strut embedment analysis by optical coherence tomography

**DOI:** 10.1007/s10554-016-0856-6

**Published:** 2016-02-22

**Authors:** Yohei Sotomi, Hiroki Tateishi, Pannipa Suwannasom, Jouke Dijkstra, Jeroen Eggermont, Shengnan Liu, Erhan Tenekecioglu, Yaping Zheng, Mohammad Abdelghani, Rafael Cavalcante, Robbert J. de Winter, Joanna J. Wykrzykowska, Yoshinobu Onuma, Patrick W. Serruys, Takeshi Kimura

**Affiliations:** Academic Medical Center, University of Amsterdam, Amsterdam, The Netherlands; ThoraxCenter, Erasmus Medical Center, Rotterdam, The Netherlands; Northern Region Heart Center, Faculty of Medicine, Chiang Mai University, Chiang Mai, Thailand; Division of Image Processing, Department of Radiology, Leiden University Medical Center, Leiden, The Netherlands; International Centre for Circulatory Health, NHLI, Imperial College London, London, UK; Department of Cardiovascular Medicine, Kyoto University Hospital, Kyoto, Japan

**Keywords:** Strut embedment, Polymeric scaffold, Metallic stent, Reproducibility

## Abstract

The degree of stent/scaffold embedment could be a surrogate parameter of the vessel wall-stent/scaffold interaction and could have biological implications in the vascular response. We have developed a new specific software for the quantitative evaluation of embedment of struts by optical coherence tomography (OCT). In the present study, we described the algorithm of the embedment analysis and its reproducibility. The degree of embedment was evaluated as the ratio of the embedded part versus the whole strut height and subdivided into quartiles. The agreement and the inter- and intra-observer reproducibility were evaluated using the kappa and the interclass correlation coefficient (ICC). A total of 4 pullbacks of OCT images in 4 randomly selected coronary lesions with 3.0 × 18 mm devices [2 lesions with Absorb BVS and 2 lesions with XIENCE (both from Abbott Vascular, Santa Clara, CA, USA)] from Absorb Japan trial were evaluated by two investigators with QCU-CMS software version 4.69 (Leiden University Medical Center, Leiden, The Netherlands). Finally, 1481 polymeric struts in 174 cross-sections and 1415 metallic struts in 161 cross-sections were analyzed. Inter- and intra-observer reproducibility of quantitative measurements of embedment ratio and categorical assessment of embedment in Absorb BVS and XIENCE had excellent agreement with ICC ranging from 0.958 to 0.999 and kappa ranging from 0.850 to 0.980. The newly developed embedment software showed excellent reproducibility. Computer-assisted embedment analysis could be a feasible tool to assess the strut penetration into the vessel wall that could be a surrogate of acute injury caused by implantation of devices.

## Introduction

The advent of OCT technology with a high resolution enabled us to assess quite precisely the appearance of metallic or polymeric struts embedded in the vessel wall. The degree of embedment could be one of surrogate parameters of the vessel wall-stent/scaffold interaction after the implantation of the scaffold/stent struts [1–4]. Historically, in the era of metallic stents, the association between stretch and deep injury of the coronary artery and neointima formation was demonstrated in a porcine model [5–7]. The vessel injury is also one aspect of vessel wall-stent/scaffold interaction. Several concerns on clinical outcomes following Absorb everolimus-eluting bioresorbable scaffold [Absorb BVS] (Abbott Vascular, Santa Clara, CA, USA) implantation stem from its inherent material property (poly L-lactic acid), scaffold design, mechanical properties of the device, etc. Recent publications reported the potential association between the larger abluminal scaffold surface area (“footprint”) of the Absorb BVS with a higher incidence of peri-procedural myocardial infarction when compared to metallic stents [8, 9]. The vessel wall and stent/scaffold interaction might play a role in this result as reported by Kawamoto et al. [10]. The surface area of the Absorb BVS is 27 %, whereas that of XIENCE Cobalt chromium everolimus-eluting stent [CoCr-EES] (Abbott Vascular, Santa Clara, CA, USA) is 13 % [9]. When the same force is applied, Absorb BVS struts create less parietal pressure compared to metallic struts, which could result in less embedment of Absorb BVS struts [1]. The degree of embedment (less protrusion of the device in the lumen) also strongly influences the endothelial shear stress in the microenvironment surrounding the struts, which is associated with neointimal formation and platelet aggregation, etc. [1, 4, 11–13].

When OCT started to be applied to metallic stents and/or polymeric scaffolds, specific and appropriate methods of analysis related to each device were used and enables fair comparison between the two devices due to the light transparency of one device versus the higher opacity of the other device [14].

Reporting of the degree of embedment seems important to describe the difference in device-vessel interaction [14]. Before the era of bioresorbable scaffolds, clinical relevance of metallic stent strut embedment with neointimal coverage was evaluated [15]. However, there was no quantitative assessment of degree of strut embedment. Now we have accurate imaging technology and comparative methodology for the assessment of metallic stents and polymeric scaffolds. We have developed a new specific method for the quantitative and accurate evaluation of embedment of struts by optical coherence tomography (OCT). In the present study, we described the algorithm of the embedment analysis and its reproducibility.

## Methods

### Study subjects

A total of 4 pullbacks of OCT images in 4 randomly selected coronary lesions with 3.0 × 18 mm devices (2 lesions with 3.0 × 18 mm Absorb BVS and 2 lesions with 3.0 × 18 mm XIENCE CoCr-EES were evaluated in this analysis. These OCT pullbacks came from ABSORB Japan, a prospective, multicentre, randomized, single-blind, active-controlled clinical trial in which 400 patients were recruited in Japan. Patients were randomized in a 2:1 ratio to treatment with the Absorb BVS or the XIENCE Prime/Xpedition CoCr-EES. The details of the trial were described elsewhere [16].

### Optical coherence tomography data acquisition

OCT pullbacks were obtained at baseline after the stent or scaffold implantation by a Frequency-domain ILUMIEN OPTIS system using a Dragonfly™ Duo catheter (St. Jude Medical Inc., Saint Paul, MN, USA) with 10–15 μm axial and 20–40 μm lateral resolution [17] at a rotation speed of 180 frames/s with non-occlusive technique [18]. After infusion of intracoronary nitroglycerine, the imaging wire was withdrawn by a motorized pullback at a constant speed of 18 mm/s, while contrast was infused through the guiding catheter at a continuous rate of 2–4 mL/min. Accordingly, OCT images were obtained per 100 μm in longitudinal length.

### Development of embedment analysis by optical coherence tomography

The embedment parameters measured by the software are strut thickness, embedment strut width and embedment depth.

**Fig. 1:**
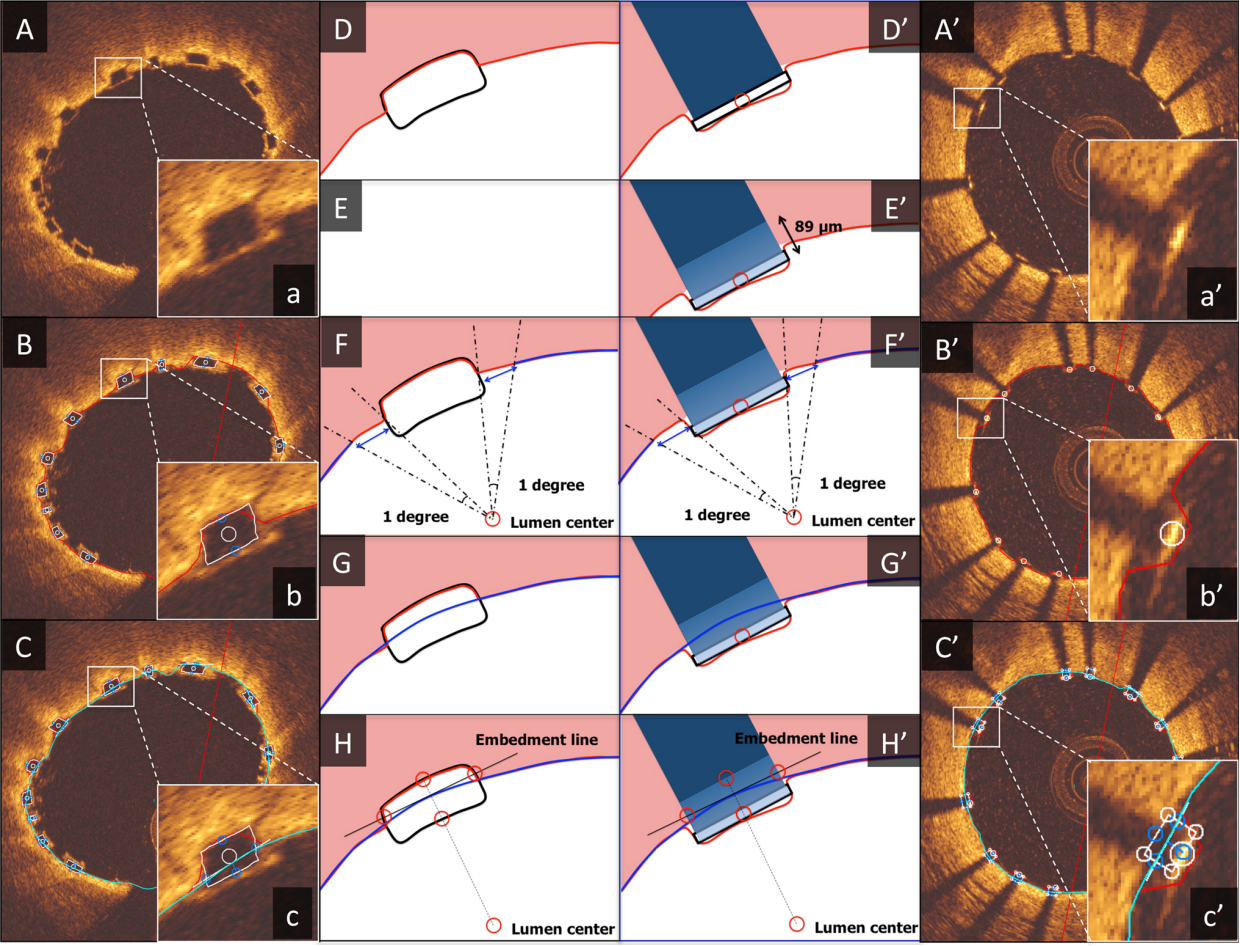
Algorithm for embedment analysis. The algorithm for embedment analysis in Absorb BVS (**A**–**H**) and XIENCE (**A**′–**H**′) is demonstrated in this figure. **A**–**C** and **A**′–**C**′ indicate the actual analysis display, **a**–**c** and **a**′–**c**′ show the magnified views of a single strut, and **D**–**H** and **D**′–**H**′ illustrate the step-by-step algorithm for embedment analysis. As a first step, automatic lumen contour detection and automatic strut detection were performed (**D**, **D**′). After detection of the abluminal side of the metallic struts, the entire body of the strut was automatically drawn by simulating the virtual contour of the struts using the thickness of the strut indicated by the manufacturer (XIENCE: 89 μm) (**E**′). The following steps were the same between Absorb BVS and XIENCE. After erasing a part of the lumen contour surrounding a strut (strut part and bilateral 1 degree measured from the lumen center) (**F**, **F**′), interpolated lumen lines were connected through the strut automatically (**G**, **G**′). “Embedment Line” was automatically delineated as described in the main text (**H**, **H**′). This additional line was used for embedment analysis to compute the following embedment measurements. “Embedment depth” was the distance between the back position of struts and the Embedment Line measured along the line from the back position through the lumen center. “Embedment strut width” was the distance between the intersection point(s) of the Embedment Line and the strut contour

In the polymeric scaffold (Absorb BVS), its black core was framed by a light reflecting structure of 30 μm (layer of the amorphous polylactide containing and releasing everolimus) (Fig. 1). Therefore, actual strut thickness of Absorb BVS was calculated as follows: Corrected Strut Thickness = strut thickness (black core thickness) + 0.06 mm [2 × 30 μm (the thickness of bright border)]. Actual embedment depth of Absorb BVS was also corrected as: Corrected embedment depth = embedment depth + 0.03 mm (the thickness of abluminal bright border). Actual embedment strut width was calculated as follows: Corrected embedment strut width = width of strut (black core) + 0.06 mm [2 × 30 μm (the thickness of bright border for both sides). In the metallic stent (XIENCE), no additional correction was performed. In the following sentences, “strut thickness”, “embedment depth” and “embedment strut width” are corrected in case of Absorb BVS and non-corrected in case of XIENCE, respectively.

**Fig. 2:**
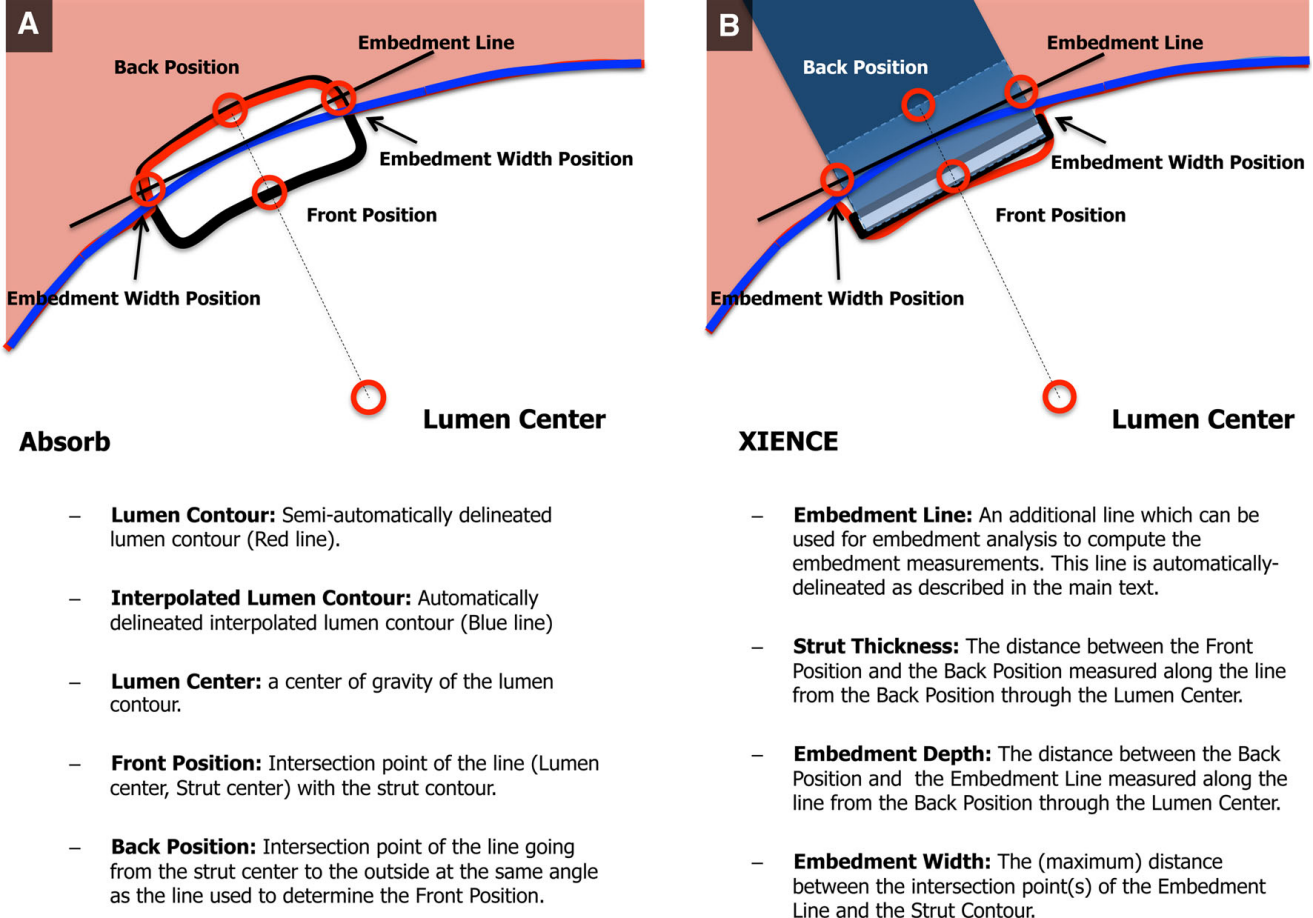
Parameters for embedment analysis. Parameters of embedment analysis for Absorb BVS (**A**) and XIENCE (**B**)

The parameters evaluated in the embedment analysis are demonstrated in Fig. 2. The “embedment ratio” (degree of embedment in percentage) was calculated using the following formula: embedment depth (the distance between the mid-point of the abluminal strut border to the interpolated lumen contour)/the thickness of the strut × 100 (%).

**Fig. 3:**
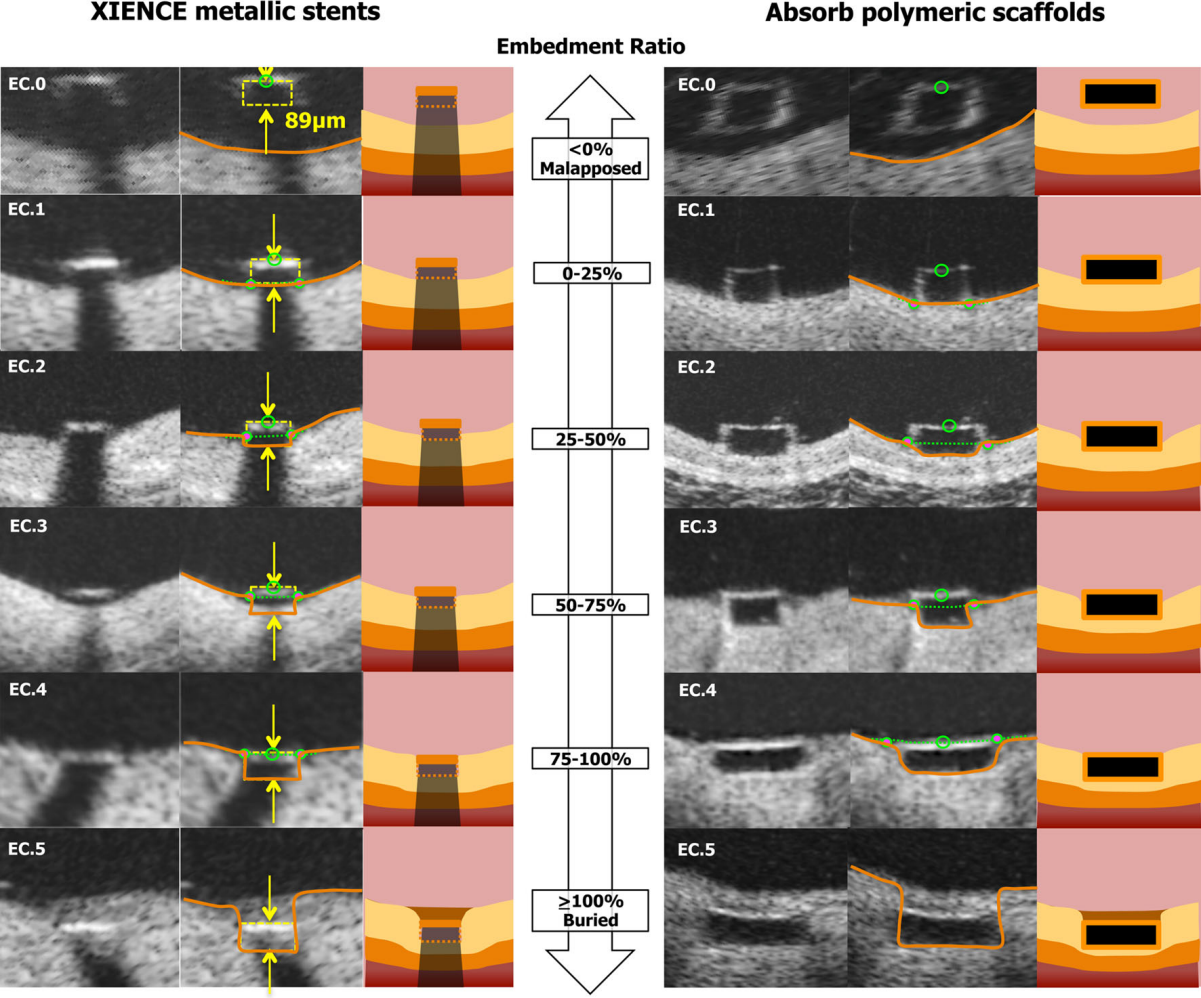
Embedment categorization. The embedment of struts was classified into 6 classes (Embedment Class [EC] 0–5) based on the degree of embedment (percentage). If struts were malapposed (indicated as negative value of percentage in the software), this was classified as EC0. When the strut was partially embedded in the vessel wall, the degree of embedment was categorized by each quartile (0 % ≤ EC1 < 25 %. 25 % ≤ EC2 < 50 %, 50 % ≤ EC3 < 75 %, 75 % ≤ EC4 < 100 %). When the tissue was covering the endoluminal surface of struts, the struts were considered as “buried”, EC5 (≥100 %)

The embedment of struts was classified into 6 classes (Embedment Class [EC] 0–5) based on the degree of embedment (percentage) as indicated in Fig. 3. If struts were malapposed (indicated as negative value of percentage in the software), this was classified as EC0. When the strut was partially embedded in the vessel wall, the degree of embedment was categorized by each quartile (0 % ≤ EC1 < 25 %. 25 % ≤ EC2 < 50 %, 50 % ≤ EC3 < 75 %, 75 % ≤ EC4 < 100 %). When the tissue was covering the endoluminal surface of struts, the struts were considered as “buried”, EC5 (≥100 %).

### Embedment analysis algorithm

All the OCT analysis was performed with a special version of QCU-CMS version 4.69 (Leiden University Medical Center, Leiden, The Netherlands). The OCT analysis was performed every 200 μm cross-section in the stent/scaffold segments. All struts from both investigators were completely matched before the assessment of embedment. Struts located at a side branch ostium were excluded from the embedment analysis. The algorithm for embedment analysis is illustrated in the Fig. 1. At the first step, automatic lumen contour detection and automatic strut detection were performed. The details of the strut detection algorithm are described elsewhere [19, 20]. For the polymeric scaffold (Absorb BVS), the black core of struts were delineated using automatic detection, and if necessary manually corrected. For the metallic stents, the center of the reflective border of the metallic strut was detected automatically by the software. If the automatically detected strut point was not located at the correct point, manual correction was performed additionally. The abluminal side of the metallic struts could not be directly delineated; however, this could be automatically drawn by simulating the virtual contour of the struts using the thickness of the strut indicated by the manufacturer (XIENCE: 89 μm). The following steps were the same for Absorb BVS and XIENCE. First, the parts of the lumen contour surrounding a strut (the strut part plus 1 degree to both sides of a strut from the lumen center) were removed and, using spline interpolation, a new interpolated lumen was automatically computed. The lumen center was detected automatically as a center of gravity of the lumen contour. Next, for each strut an “Embedment Line” was computed automatically as follows: based on the intersection of the interpolated lumen contour with lines from the lumen center through the start/end angle of each strut plus 2.5° to each side, an intersection line was computed. This intersection line was then moved to touch the interpolated lumen along a line from the lumen center through the center of the intersection line. This additional line was used for embedment analysis to compute the following embedment measurements. “Embedment depth” was the distance between the back position and the embedment line measured along the line from the back position through the lumen center. “Embedment strut width” was the largest distance between the intersection point(s) of the embedment line with the strut contour. The embedment strut width was evaluated only when the embedment line intersected the strut contour. If there was no intersection between the embedment line and the strut contour, embedment strut width was not analyzed.

### Assessment of reproducibility

For the assessment of intra- and inter-observer reproducibility, two analysts (Observer A, HT and Observer B, YS) performed OCT embedment analysis. For the intra-observer reproducibility, one of the analysts (YS) repeated all the measurements on the same pullbacks after an interval of 4 weeks. For the evaluation of inter-observer reproducibility, the parameters of strut embedment were compared between the two analysts. The agreement between the two analysts for the embedment categorization was also determined.

### Statistical analysis

Quantitative measurements to assess the inter- and intra-observer reproducibility are presented at strut level analysis. Data are expressed as mean ± standard deviation or median and inter-quartile range, if appropriate. Intra- and inter-observer reproducibility was evaluated by the following methods. The reproducibility of embedment parameter measurements (embedment ratio and embedment strut width) at strut level was evaluated with the interclass correlation coefficient (ICC) for concordance (ICCc) and absolute agreement (ICCa) with its 95 % confidence intervals (CI). An ICC < 0.4 indicates bad agreement, an ICC between 0.4 and 0.75 indicates moderate agreement, and ICC values > 0.75 indicates excellent agreement [21]. The correlation between different observations was analyzed by simple linear regression. Measurement agreement was determined by comparing measurements of each analysis using the Bland–Altman method [22]. Data are given as plots showing the absolute difference between corresponding measurements of both observers (y-axis) against the average of both observers (x-axis). The relative difference between measurements (absolute difference divided by the average) gives the bias; its standard deviation gives the random variation. The limits of agreement were calculated as mean bias ± 1.96SD. The Cohen’s **κ** (kappa) test was used to assess intra- and inter-observer agreement for embedment categorization. The kappa coefficient was categorized as <0.20 = poor, 0.21–0.40 = fair, 0.41–0.60 = moderate, 0.61–0.80 = substantial, and 0.81–1.00 = almost perfect [23]. Statistical significance was assumed at a probability (*P*) value of <0.05. All statistical analyses were performed with SPSS (version 23.0.0, IBM, New York) and MedCalc Statistical Software version 14.12.0 (MedCalc Software bvba, Ostend, Belgium).

## Results

### Population characteristics

A total of 177 and 188 cross-sections were recognized in the scaffolded and stented segments, respectively. In 3 of 177 and 27 of 188 cross-sections, automatic lumen detection did not work appropriately due to poor image quality. In the remaining 174 and 161 cross-sections, 1481 polymeric struts and 1415 metallic struts were matched and analyzed for embedment assessment. The embedment analysis for one case took on average 25 ± 6 min for 18 mm device with 200 μm intervals (theoretically 90 cross-sections). We performed manual correction in 3.9 ± 0.7 % of all the struts.

### Reproducibility of quantitative measurements

**Fig. 4:**
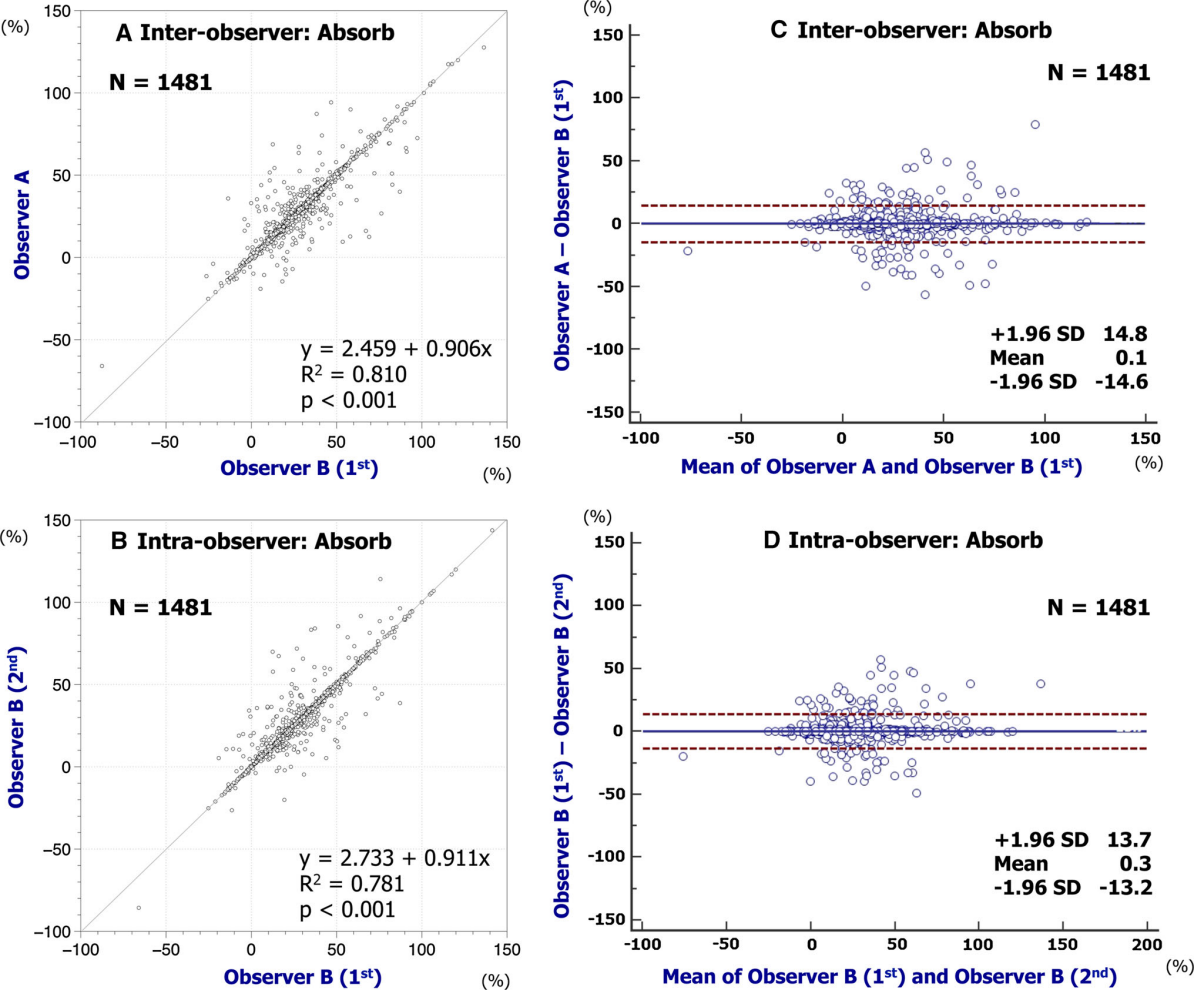
Reproducibility for embedment ratio of Absorb BVS. Simple linear regression analyses are indicated in **A** (inter-) and **B** (intra-observer). Bland–Altman plots indicate inter- (**C**) and intra-observer (**D**) reproducibility to assess the embedment ratio of Absorb BVS

**Fig. 5:**
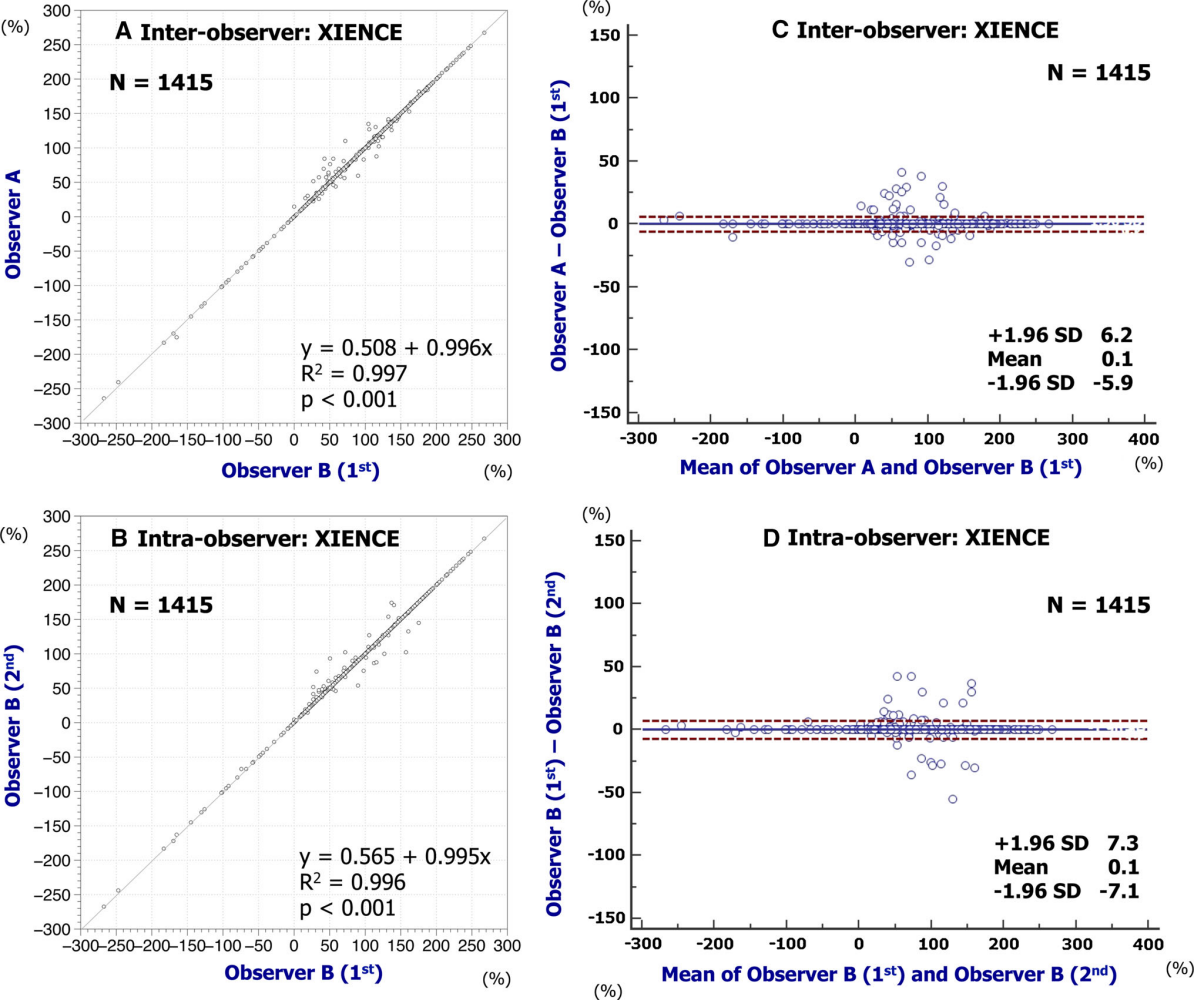
Reproducibility for embedment ratio of XIENCE. Simple linear regression analyses are indicated in **A** (inter-) and **B** (intra-observer). Bland–Altman plots indicate inter- (**C**) and intra-observer (**D**) reproducibility to assess the embedment ratio of XIENCE

**Fig. 6:**
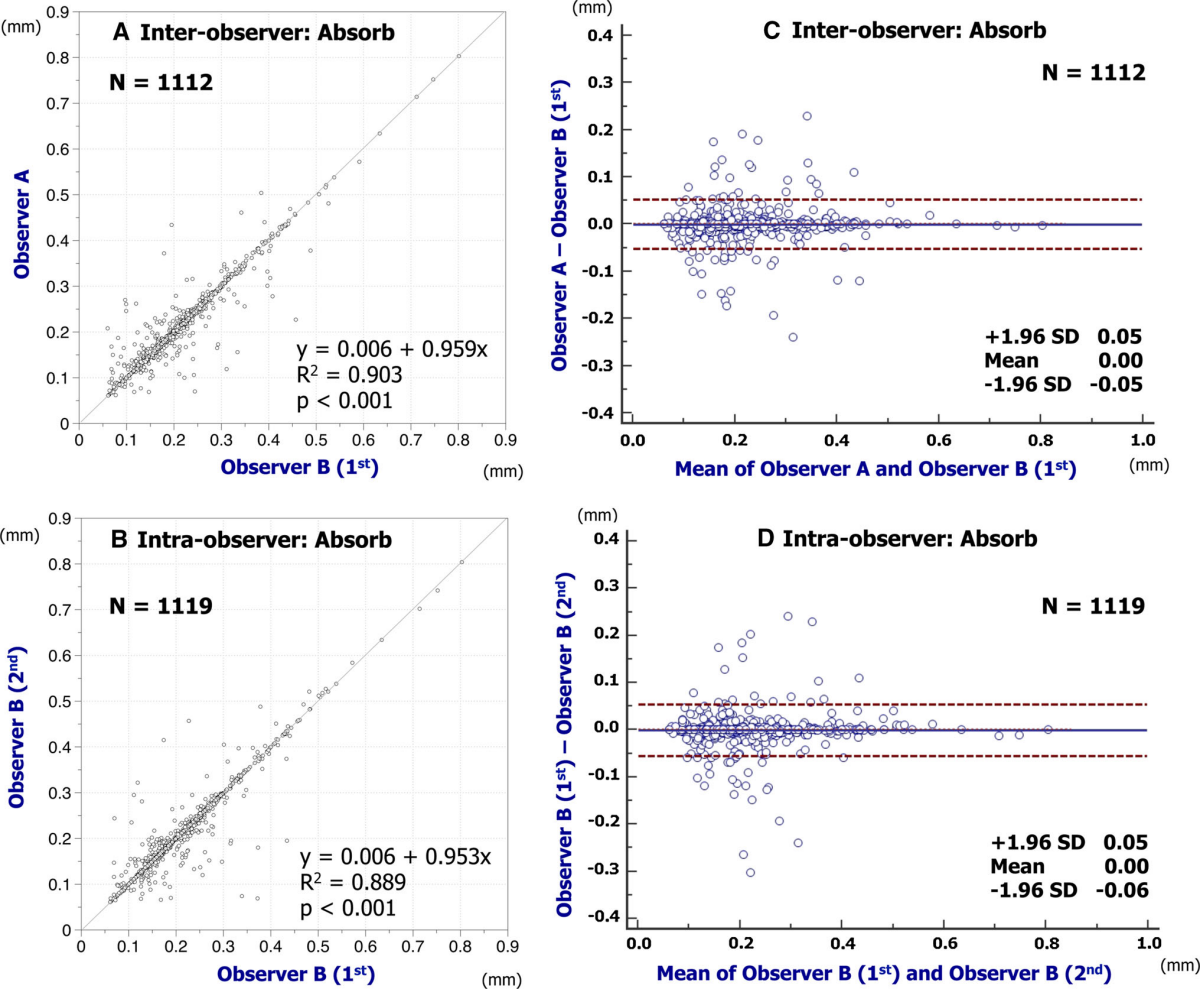
Reproducibility for embedment strut width of Absorb BVS. Simple linear regression analyses are indicated in **A** (inter-) and **B** (intra-observer). Bland–Altman plots indicate inter- (**C**) and intra-observer (**D**) reproducibility to assess the embedment strut width of Absorb BVS

**Fig. 7:**
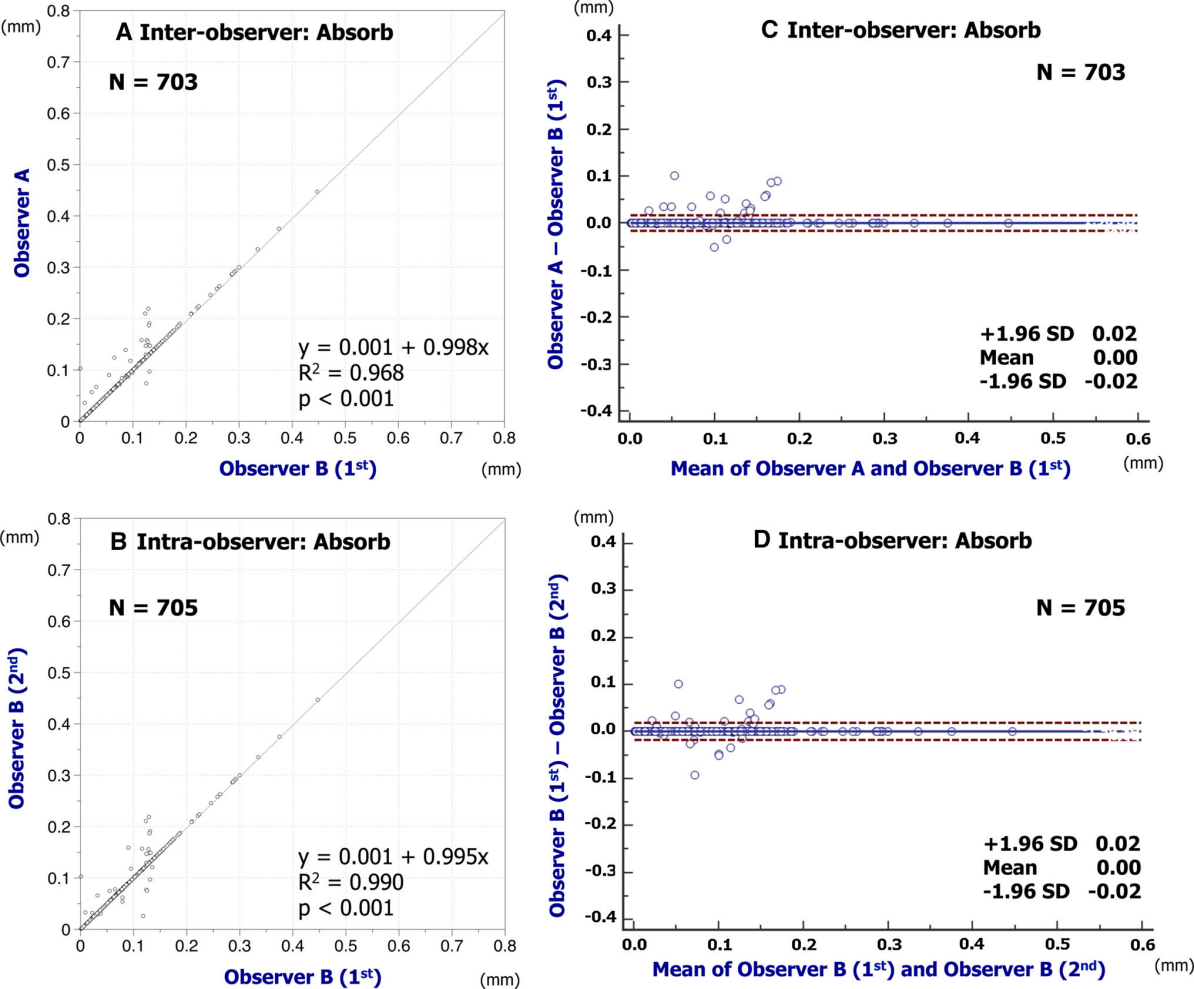
Reproducibility for embedment strut width of XIENCE. Simple linear regression analyses are indicated in **A** (inter-) and **B** (intra-observer). Bland-Altman plots indicate inter- (**C**) and intra-observer (**D**) reproducibility to assess the embedment strut width of XIENCE

**Fig. 8:**
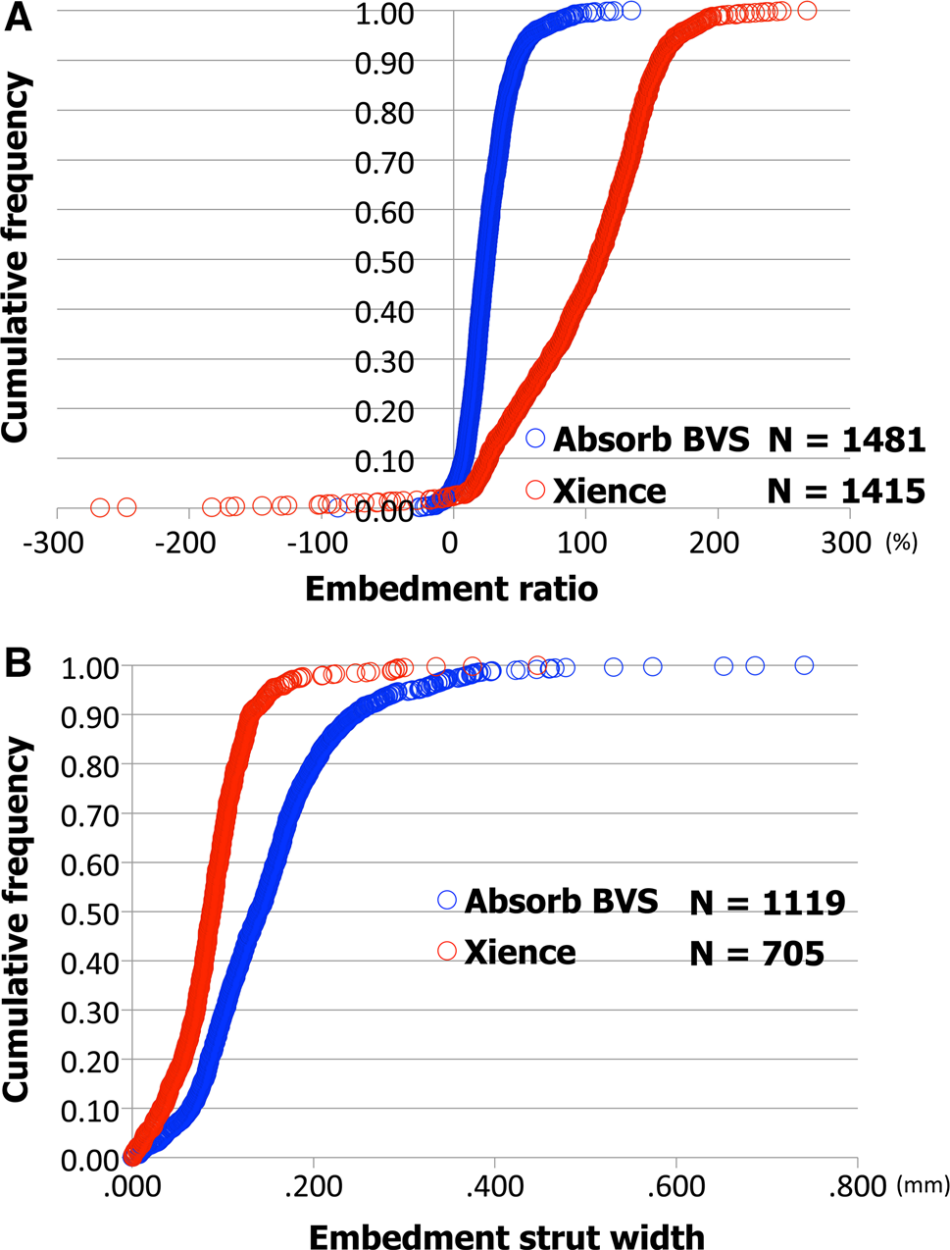
Cumulative frequency distribution curves. Cumulative frequency distribution curves of embedment ratio (**A**) and embedment strut width (**B**) assessed by observer B (1st)

Inter- and intra-observer reproducibility of quantitative measures are shown in Table 1. The assessments of embedment ratio in Absorb BVS and XIENCE had excellent agreement in both inter- and intra-observer reproducibility (Absorb BVS: inter-observer ICCc of multiple raters, 0.958 [95 % confidence interval 0.954–0.962]; intra-observer ICCc of multiple raters, 0.965 [0.962–0.969]; XIENCE: inter-observer ICCc of multiple raters, 0.999 [0.999–0.999]; intra-observer ICCc of multiple raters, 0.999 [0.999–0.999]). The assessments of embedment strut width in Absorb BVS and XIENCE also had excellent agreement in both inter- and intra-observer reproducibility (Absorb BVS: inter-observer ICCc of multiple raters, 0.974 [0.971–0.977]; intra-observer ICCc of multiple raters, 0.971 [0.968–0.974]; XIENCE: inter-observer ICCc of multiple raters, 0.992 [0.991–0.993]; intra-observer ICCc of multiple raters, 0.991 [0.989–0.992]). Simple linear regression and Bland–Altman plots for embedment ratio and embedment strut width are shown in Figs. 4, 5, 6, 7. Cumulative frequency distribution curves of embedment ratio and embedment strut width are indicated in Fig. 8.

**Table 1:** Inter- and intra-observer reproducibility of quantitative measures

	No. of matched struts	Inter-observer variability	Observer A versus observer B (1st)	ICCc	ICCa
		Analyzable matched struts	Absolute difference [95 % CI]		
*Embedment ratio (%)*					
Absorb BVS	1481	1481	−0.08 [−0.51–0.36]		
Single measures				0.919 [0.911–0.927]	0.919 [0.911–0.927]
Average measures				0.958 [0.954–0.962]	0.958 [0.954–0.962]
XIENCE	1415	1415	0.14 [−0.02–0.30]		
Single measures				0.998 [0.998–0.999]	0.998 [0.998–0.999]
Average measures				0.999 [0.999–0.999]	0.999 [0.999–0.999]
*Strut embedment width (mm)*					
Absorb BVS	1481	1112	−0.000 [−0.002–0.002]		
Single measures				0.95 [0.943–0.955]	0.95 [0.943–0.955]
Average measures				0.974 [0.971–0.977]	0.974 [0.971–0.977]
XIENCE	1426	703	0.001 [0.000–0.002]		
Single measures				0.984 [0.981–0.986]	0.984 [0.981–0.986]
Average measures				0.992 [0.991–0.993]	0.992 [0.990–0.993]

	No. of matched struts	Intra-observer variability	Observer B (1st) versus observer B (2nd)	ICCc	ICCa
		Analyzable matched struts	Absolute difference [95 % CI]		
*Embedment ratio (%)*					
Absorb BVS	1481	1481	−0.33 [−0.80–0.15]		
Single measures				0.933 [0.926–0.939]	0.933 [0.926–0.939]
Average measures				0.965 [0.962–0.969]	0.965 [0.962–0.969]
XIENCE	1415	1415	0.08 [−1.09–0.27]	0.998 [0.998–0.999]	0.998 [0.998–0.998]
Single measures				0.999 [0.999–0.999]	0.999 [0.999–0.999]
Average measures					
*Strut embedment width (mm)*					
Absorb BVS	1481	1119	0.001 [−0.001–0.002]		
Single measures				0.944 [0.937–0.95]	0.944 [0.937–0.95]
Average Measures				0.971 [0.968–0.974]	0.971 [0.968–0.974]
XIENCE	1426	705	0.001 [−0.000–0.001]		
Single measures				0.982 [0.979–0.984]	0.982 [0.979–0.984]
Average measures				0.991 [0.989–0.992]	0.991 [0.989–0.992]

### Reproducibility of qualitative measurements

The inter- and intra-observer reproducibility of embedment category at strut level analysis is shown in Table 2. Inter- and intra-observer reproducibility to assess embedment category was very good both in Absorb BVS (inter-observer kappa, 0.850; intra-observer kappa, 0.867) and XIENCE (inter-observer kappa, 0.976; intra-observer kappa, 0.980), but better in the XIENCE than in the Absorb BVS.

**Table 2:** The inter- and intra-observer reproducibility of embedment category

	Observer A						Total	Inter-observer agreement (Kappa)
	Embedment category							
	0	1	2	3	4	5		
*Observer B (1st)*								
Absorb BVS								
Embedment category								
0	54	11	0	0	0	0	65	0.850
1	8	652	33	4	0	0	697	
2	2	40	534	7	3	0	586	
3	0	3	12	80	6	1	102	
4	0	0	2	3	19	1	25	
5	0	0	0	0	0	6	6	
Total	64	706	581	94	28	8	1481	
XIENCE								
Embedment category								
0	33	0	0	0	0	0	33	0.976
1	0	96	2	0	0	0	98	
2	0	1	149	8	1	0	159	
3	0	0	2	145	3	1	151	
4	0	0	0	2	184	0	186	
5	0	0	0	0	2	786	788	
Total	33	97	153	155	190	787	1415	

	Observer B (2nd)						Total	Intra-observer agreement (Kappa)
	Embedment category							
	0	1	2	3	4	5		
*Observer B (1st)*								
Absorb BVS								
Embedment category								
0	53	10	2	0	0	0	65	0.867
1	6	656	30	5	0	0	697	
2	1	31	543	9	2	0	586	
3	0	3	14	80	5	0	102	
4	0	0	2	0	22	1	25	
5	0	0	0	0	0	6	6	
Total	60	700	591	94	29	7	1481	
XIENCE								
Embedment category								
0	33	0	0	0	0	0	33	0.98
1	0	97	1	0	0	0	98	
2	0	0	154	5	0	0	159	
3	0	0	3	144	3	1	151	
4	0	0	0	1	185	0	186	
5	0	0	0	0	4	784	788	
Total	33	97	158	150	192	785	1415	

## Discussion

The present study demonstrated a high reproducibility for in vivo quantitative assessment of scaffold/stent embedment by OCT. The assessments of embedment ratio in Absorb BVS and XIENCE had excellent agreement in both inter- and intra-observer reproducibility. Inter- and intra-observer reproducibility to assess embedment category was also very good both in Absorb BVS and XIENCE. The algorithm and semi-automatic program for embedment analysis was reproducible and appeared to be feasible to use in future studies.

### Clinical application of embedment analysis

Before the era of OCT, namely in the era of metallic stents, angiography and intravascular ultra sound, there was no accurate assessment of embedment. The scientific interest for embedment came from the need for accurate and quantitative evaluation of the vessel wall and stent/scaffold interaction. The previous animal studies on histology indicated a clear relationship between injury and neointimal proliferation [5–7]. The assessment of embedment on OCT could have been a surrogate parameter of vessel wall injury in these early days [1].

Our results indicated that the boundary of agreement in the continuous value of embedment ratio was as narrow as 15 %; and the kappa value in the embedment category was as high as 0.850, which may allow us to use continuous values or categories of embedment for scientific purpose. From a practical point of view, we can also use OCT embedment assessment to evaluate the quality of stent/scaffold implantation. We would be able to express the results in percentage of embedment and, as usual, we would have strut level assessment, cross sectional level assessment, and scaffold/lesion level assessment.

### Embedment strut width and vessel-stent/scaffold interaction

The width of the strut could also influence the embedment of the strut. When the same force is applied, a device with a smaller contact area would generate a higher pressure to the vessel wall according to the simple principle: Pressure = Force/Area, resulting in more embedded struts. Embedded struts denote penetration of the cutting edge of the struts through fibrous, calcific, and necrotic plaques, implying larger injury of the vessel. On the other hand, Kawamoto et al. reported the potential association between the larger footprint of the Absorb BVS and higher incidence of peri-procedural myocardial infarction when compared with metallic stent [8, 9]. Even if the embedment of struts is small, a larger footprint (larger width of struts) itself could contribute to larger amount of vessel wall-stent/scaffold interaction.

The relationship amongst embedment depth, embedment strut width, and vessel injury will be a topic of research in the upcoming year [1]. Our algorithm demonstrated excellent reproducibility for the assessment of embedment strut width as well.

### Advantages and disadvantages of embedment

Whatever is embedded in the vessel wall does not impact the flow in the lumen. Flow area increases as embedment increases; and in terms of shear stress, the deeper the struts are embedded, the less disturbed the shear stress will be [13]. However, there seems to be a down side in the sense that the embedment might also be the expression of a kind of injury that can trigger the neointimal hyperplasia as a response to injury [5–7]. Eluted cytotoxic and cytostatic drugs have been introduced to inhibit the excessive neointimal formation. On that theoretical basis, we should not expect an excess of neointima despite the embedment and injury to the vessel wall. The relationship between the injury (degree of embedment) and neointimal hyperplasia will be the topic of future studies.

## Limitation

Selection bias of the patients and cross-sections was the major limitation of this analysis. The sample size of the enrolled patients was quite limited, although the strut number was sufficient to evaluate the reproducibility of the method. A total of 30 cross-sections from 365 cross-sections were excluded from the analysis due to incapability of automatic lumen detection (masked by residual blood). This automatic detection, a key factor for the excellent reproducibility of this embedment algorithm, was highly influenced by the OCT image quality. Some sample showed as much as 50 % of difference in embedment ratio. These differences stemmed from the struts and lumen contours manually corrected by analysts. In some struts and lumen contours, we needed to manually correct the strut point and contours because of the error of the automatic detection [19, 20]. Although we have created a protocol for manual correction to improve the reproducibility as much as possible, this kind of manual work affected the accuracy of the analysis. Finally, in the current study, we focused only on the embedment analysis and its reproducibility, which is just one aspect of vessel injury assessment. Further investigation would be necessary to assess the vessel injury comprehensively.

## Conclusions

The newly developed embedment analysis by OCT showed excellent reproducibility in stented/scaffolded coronary segments. Computer-assisted embedment analysis could be a feasible tool for future clinical application and clinical studies.
